# Low loaded Pt-Co catalyst surfaces optimized by magnetron sputtering sequential deposition technique for PEM fuel cell applications: physical and electrochemical analysis on carbon paper support

**DOI:** 10.3906/kim-2101-50

**Published:** 2021-10-19

**Authors:** Osman ÖZTÜRK, Aydın HAŞİMOĞLU, Oğuz Kaan ÖZDEMİR, İnci KARAASLAN, Ali Şems AHSEN

**Affiliations:** 1 Department of Physics, Gebze Technical University, Kocaeli Turkey; 2 Nanotechnology Research Center, Gebze Technical University, Kocaeli Turkey; 3 Department of Metallurgical and Material Engineering, Yıldız Technical University, İstanbul Turkey; 4 Department of Material Science and Engineering, Gebze Technical University, Kocaeli Turkey; 5 Department of Chemistry, Gebze Technical University, Kocaeli Turkey

**Keywords:** Proton exchange membrane fuel cell, magnetron sputtering, Pt-Co catalyst, X-ray photoelectron spectroscopy, cyclic voltammetry

## Abstract

A series of thin Pt-Co films with different metal ratios were deposited by using the sequential cosputtering directly on a commercial hydrophobic carbon paper substrate at room temperature and in ultra-high vacuum (UHV) conditions. Their electrocatalytic properties toward the oxygen reduction reaction were investigated in 0.5 M H_2_SO_4_ solution by means of cyclic voltammetry (CV) and linear sweep voltammetry (LSV) on rotating disc electrode (RDE). The results showed that Pt particles, deposited by dc-magnetron gun, surround the large Co-clusters deposited by rf-magnetron gun. In addition, the increase of Co content led to an increase in the electrochemical active surface area (EASA) from 23.75 m^2^/gPt to 47.54 m^2^/gPt for pure Pt and Pt:Co (1:3), respectively, which corresponded the improvement of the utilization of Pt by a factor of 1.91. This improvement indicated that the sequential magnetron cosputtering was one of the essential technique to deposit homogeneous metal clusters with desirable size on the gas diffusion layer by adjustment plasma parameters.

## 1. Introduction

Fuel cells (FC) have been receiving significant attention in the recent decades due to their high efficiency and environmental compatibility [1–2]. Among all fuel cell systems, proton exchange membrane fuel cells (PEMFCs) have shown a great promise as an alternative power source, particularly for stationary power generation and transportation applications because of their low operating temperature, fast start-up, high power density, and low emission of pollutants [3]. Although there have been some serious improvements in PEMFCs, they are still not widely used as their cost and durability are unsatisfactory. These are considered as the most serious impediments for the commercialization of PEMFCs [4]. 

There are many potential losses during the FC operation such as the mass transport loss and the ohmic loss as well as the oxygen reduction reaction (ORR) kinetic loss. Most of the researches on the PEMFCs were focused on improving the catalytic activity of the cathode electro-catalysts, where the ORR takes place. ORR slow rates were responsible for about 70% of the total losses that occur in PEMFCs [5–6]. The common approach to develop electrocatalysts for PEMFCs is to investigate their activities (using CV experiments) against ORR and hydrogen oxidation reaction (HOR) in a three-electrode cell, which is filled with an oxygen saturated acid electrolyte. Here, the catalyst coated diffusion media was at the focused point and platinum have been regarded as the most active catalyst for both HOR and ORR. On the other hand, at the two electrode sides, the electro-catalyst reactions struggled with corrosion kinetic, platinum dissolution, and redeposition [7]. Hubert et al. showed the significant increase of MEA performance was related to decrease in the mass transport-induced voltage losses combined with the reduction of Pt [8, 9]. Some of reviews suggested the Pt-based alloys on the enhancement of the kinetics in order to create the enveloped non-Pt metal by Pt against the aggressive chemical environment [10, 11]. In addition to performance improvements, a decrease in catalytic loading was highly desirable for fuel cell cost reduction. Although the Pt-based alloy electro-catalysts were the most used ones to improve electro-catalytic activity for ORR, it was observed that the electro-catalyst performance strongly depends on the electrode fabrication method. 

In order to reduce the amount of Pt in PEMFCs, usually obtained as nanoparticles, Pt was often alloyed with transition metals such as Fe, Co, Ni, Cr, etc. at different Pt:M ratios [12]. Some Pt-based alloys have demonstrated higher electrocatalytic activity toward ORR than pure Pt. The improvement in ORR efficiency has been attributed to structural and/or electronic effects caused by alloying. In particular, Pt-Co alloy showed the highest ORR activity among Pt-M alloys [13, 14]. Although Pt-Co alloys have been shown to provided 1–2.5 gain of electrochemical activity (mass activity) for oxygen reduction reaction compared to pure Pt, the long-term stability of Pt-M alloys under the working conditions of PEMFCs was still not satisfactory [15–21]. The observed decay in the performance over long service life was explained by the loss of active surface area due to particle agglomeration on the catalyst surface or by leaching of the nonprecious metal to the electrolyte.

One of the ways to increase the efficiency of the catalyst and to reduce the Pt loading was to realize a deposition technique with the different approach. Particularly, for the thin film catalyst surfaces, the self-motivation growth process was the most dominant mechanism if the UHV thin film deposition technique was used such as magnetron sputtering. The aim was to design a surface occurred with the core-shell type electrocatalysts consisting of monolayer or submonolayer of Pt atoms enclosed the nonprecious metal. Over the past few years, the research on nanoparticles with core-shell structures has become a prevalent trend because of their lower cost, potential structural and/or electronic effects, and superior catalytic properties [22, 23]. There were various promising methods to prepare carbon supported Pt-M core-shell nanoparticles such as electroless deposition method [24, 25], ethylene glycol-assisted polyol method, co-reduction and seed growth method [26]. Among them, the magnetron sputtering deposition technique was not common for preparing heterogeneous catalysis, even not for core-shell structure. On the other hand, it was well suited to prepare high surface area nano-porous catalyst surfaces [27–31]. Meanwhile, some published works proved that the magnetron sputtering deposition had promising potential to build the catalyst surface containing catalyst particles like core-shell structures [32]. Mani et al. reported that the surface having core-shell type catalyst particles showed the surface catalytic activities improved four times higher than the pure Pt surface in the cathode of PEMFC [33]. Although the focus in most research were about developing wet alloying techniques to improve catalytic activities in PEMFCs, in present study, the magnetron co-sputtering deposition technique was used to increase the effectiveness of the catalyzer thin film surfaces. The synthesis techniques in UHV such as magnetron sputtering provided limited control on the surface, since the self-growing process developed on the substrate. Therefore, the sensitivity during the deposition and the in-situ characterization became very essential. In addition, the characterization of the growth mode, the electronic structure and the surface topology were necessary in order to observe the self-growth process.

The present study was a part of our ongoing research on the bimetal catalyst surface feasibility. The first published study was about developing Pt-Co catalyst on the glassy carbon substrate used to minimizing the effects due to surface roughness on substrate [34]. Uzunoglu et al. showed that Pt:Co (1:1) had the highest ORR performance with a mass activity of 571.17 Ag^-1^_Pt_ and a speciﬁc activity of 835.59 μAcm^-2^ among the all Co-containing catalysts, and these were different than the results obtained present study, where the same preparation conditions were used in the same magnetron sputtering chambers. In this published study, another main conclusion was that Pt particles seem to cover the Co particles so Co-oxide reaction was not observed. In order to investigate the details of the clustering Pt and Co particles, another collaborative study was published [35]. The aim was to observe the behavior of Pt and Co clusters growth on TiO_2_ (001) substrate by using e-beam deposition. Randima et al. concluded in their study that Pt clusters covered the Co clusters on the contrary what was expected based on the surface energy of Pt (2.5 J/m^2^) and Co (1.9 J/m^2^). Based on the surface energy of Pt and Co, it was expected that Pt particles should have covered by Co particles or penetrate each other at least on the surface of the particles, but it did not. Both published works revealed that the performance of Pt-Co thin ﬁlm catalysts deposited on ﬂat surfaces highly depends upon the sputtering temperatures, surface morphology, the composition of the ﬁrst few atomic layers, and the stability of the catalyst layers. It should be noted here that the substrates for both studies are almost well defined flat surfaces (glassy carbon and Titanium Dioxide) unlike carbon paper. Therefore, it is meaningful to characterize Pt-Co catalyst layer on carbon paper, which is used as a gas diffusion layer in PEMFC.

 In this study, Pt-Co thin films with ultra-low loading and varying metal ratios were deposited by dc and rf magnetron sputtering and investigated as catalysts for the oxygen reduction reaction. The study aimed to find the role of Co content on the film structure, electrochemically active surface area and ORR catalytic activity in sulphuric acid solutions. 

## 2. Experimental

Thin films of Pt-Co catalyst layers were deposited on carbon paper substrate using dc and rf sputtering technique, respectively. The preparation chamber was equipped with BesTec 222 UHV magnetron sputtering system, which had a base pressure of 5 × 10^–9^ mbar. During the deposition, the pressure was in the range of 1.4 × 10^–3^ and 1.5 × 10^–3^ mbar and Ar plasma was formed using high purity Ar gas (99.99%). Target to substrate distance was set to 65 mm for both of Pt and Co. The deposition rate and thickness of each material could be monitored in situ by quartz crystal microbalance (QCM). Using the magnetron sputter technique with QCM in an UHV system enabled to prepare high purity and precise controlled ultra-thin single metal or bimetallic films. 

The catalytic surfaces of Pt-Co were growth by using sequential deposition of Pt and Co at room temperature. The magnetron sputter guns loaded with Pt and Co target, which had 3 inches radius and 99.99% purity, were fired at the same time. In the magnetron deposition chamber, the dc- and rf- power dual guns had ability to be fired the plasma at the same time for the sequential deposition. The rf-power was used for the cobalt deposition, since it was hard ferromagnetic material. Instead of alloyed target, the single element target materials were used to control the stoichiometric ratio for the deposited film. There would be used other technique such as a confocal deposition for alloying films, but the current setup in the magnetron deposition chamber did not have the properties of confocal the magnetron sources. That is why the sequential deposition was used and for every sequence, the deposition thickness was hold less than half-mono layer by controlling deposition time. The manipulator with the sample holder moved back and forth under the two sources of Pt and Co. Base on the ratio of Pt:Co and total metal amount, the cycle number and the duration for each cycle of Pt and Co were applied to computerized motion so that the last layer and first layer were deposited with Pt. The total metal loading of all samples were 22 μg.cm^–2^ where the ratio of Pt:Co were 3:1, 1:1 and 1:3. 

As a substrate, commercial carbon paper (I3/C1, Freudenberg) was used that its one side was covered with a Vulcan XC-72 microporous film and had hydrophobic treatment. The carbon paper was prepared as 5 cm squares and placed in the middle of the sample holder in order to avoid any inhomogeneity during the sputter deposition. The analysis chamber was equipped with SPECS X-Ray source XR 50 (MgKα), SPECS Phoibos 150 charged particle analyzer and SPECS Aarhus 150 SPM. The surface homogeneity was checked by both using scanning electron microscopy (STM) and scanning electron microscopy (SEM). Si (111) substrate was used to obtain high quality STM results. The wide range scan of the Pt sputtered surface showed homogenously dispersed Pt particles (see Supplementary Materials for details). The elemental composition of the catalyst surfaces was investigated using XPS technique. In addition, Pt and Co deposition rate and thickness were checked by QCM, which was installed in the magnetron sputter chamber. QCM was calibrated by using the attenuation of the Ag 3d_5/2_ XPS signal. Tanuma, Powell, and Penn’s (TPP) formula was used to calculate the amount of material on the substrate [36]. Pt and Co atomic ratios were calculated using deposition rate and element density.

The surface morphology of the sputtered Pt-Co catalyst layer on carbon paper substrate was checked via scanning electron microscopy (SEM, Philips XL30 SFEG). In addition, the wide range surface SEM/EDS mapping showed homogenously dispersed Pt-Co particles covering carbon paper substrate (see Supplementary Materials for details).

Powder X-ray diffraction (XRD) patterns of these electrocatalysts were obtained using a copper radiation source (CuKα1, λ = 0.15406 nm, Rigaku Dmax 2200) in scan-step mode from 2ϴ = 10–90°.

**Figure 1:**
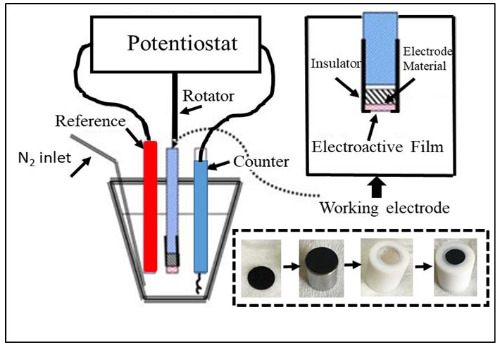
A three-electrode electrochemical cell and sample preparation for CV and LSV.

The electrochemical activities of the catalysts were investigated in electrolyte solution using basic electrochemical methods such as cyclic voltammetry (CV) and linear sweep voltammetry (LSV) with computer controlled AFCBP1 model Pine Bipotentiostat. Experimental set up used for measurements was given in Figure 1. A three-electrode electrochemical cell, which consisted of working electrode (carbon paper substrate with catalyst surface), counter electrode (Pt wire) and reference electrode (Ag/AgCl electrode), were immersed 0.5 M H_2_SO_4_ solution for electrochemical experiments. Although Ag/AgCl was used as the reference electrode, all potential values in the study corresponded to the standard hydrogen electrode. In order to complete experimental set up, a circular shaped sample was cut. Next, it was clamped in a Teflon holder with a metal piece and finally capped on the working electrode as shown in Figure 1.

 Before the electrochemical analysis all the electrodes were pre-treated for 50 cycles at scan rate of 100 mV.s^–1^ by cycling the potential in the range -0.25 V to 1.3 V to obtain reproducible data. The oxygen-saturated solution (30 min bubbling) was used for the RDE polarization curves while the nitrogen bubbled (30 min) solution was used for the CV curves. While performing both types of experiments, the relevant gas flow was kept to the electrolyte surface to prevent the atmosphere - electrolyte contact. For the RDE analysis, the potential was swept in the range from –0.12 V to + 1.0 V (vs. RHE) at scan rate of 10 mV.s^-1^ and constant rotating speed 1600 rpm. Mass transfer corrected Tafel plots were constructed with kinetic current density, which was extracted from Koutecky Levich (K–L) plots, and apparent exchange current density, j_o_^ap^, of the ORR was calculated. Integration of the area under the hydrogen adsorption/desorption peaks in cyclic voltammograms were used to evaluate the electrochemically active surface area (EASA) of the catalyst. The real exchange current density for each sample, j_o_ was calculated dividing j_o_^ap^ by EASA.

## 3. Results and discussion

**Figure 2:**
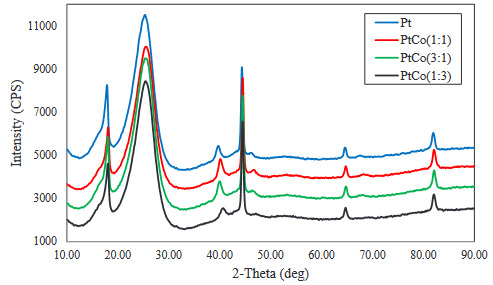
XRD diffractograms of sputtered Pt-Co layers.

**Figure 3:**
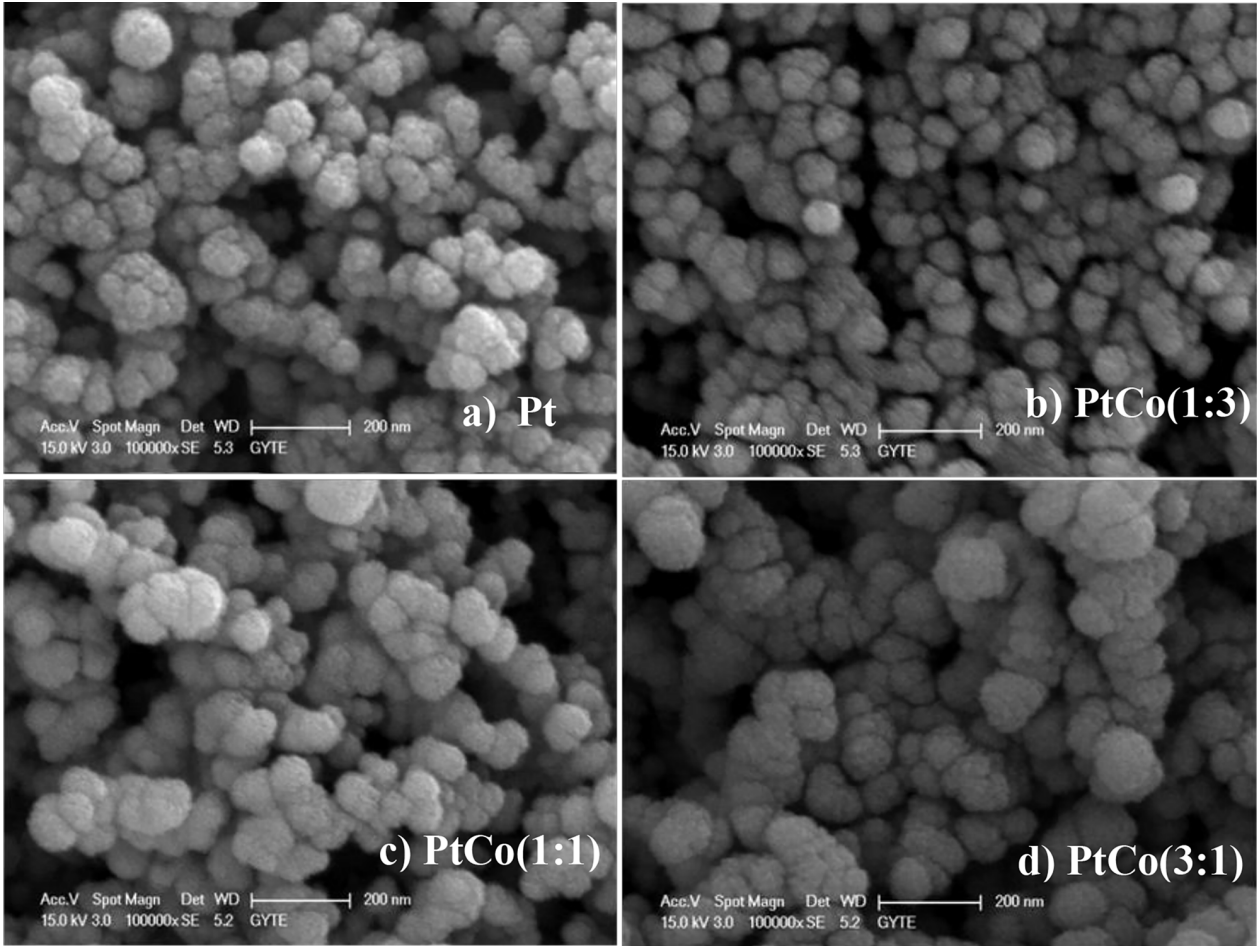
SEM images of sputtered Pt-Co layers: (a) Pt, (b) Pt:Co (1:3), (c) Pt:Co (1:1), (d) Pt:Co (3:1).

The XRD patterns of the carbon-supported Pt, Pt:Co (3:1), Pt:Co (1:1), and Pt:Co (1:3) were shown in Figure 2. The diffraction peaks at ~2ϴ = 18.0°, 24.6°, and 44.5° angles for all types of catalysts arose from the reflection of the support. All diffraction patterns displayed the typical diffraction peaks ( <111>, <220>, and <311>) of the fcc structure of platinum. No peaks were observed due to metallic Co or their oxides. There was also no noticeable shift of Pt peaks toward high angle. Particle sizes of electrocatalyst were calculated from the broadening of (111) diffraction peak in XRD pattern of all catalysts by using Scherrer equation. Particle sizes and lattice parameters of Pt were given in Table 1. It was observed that addition of Co for Pt:Co (3:1) and Pt:Co (1:1) did not change the lattice parameter significantly, but increased the particle size. On the other hand, for Pt:Co (1:3), the addition of Co in the electrocatalyst layer caused the shrink of Pt lattice and decreased the particle size because of strain caused by high amount of Co compared to Pt. SEM images of all catalysts were shown in Figure 3. In agreement with the XRD calculations, it could be seen that Pt:Co (3:1) and Pt:Co (1:1) were larger than Pt and Pt:Co (1:3). Due to higher Pt atomic percent, this phenomenon was attributed to the agglomeration of Pt particles. These observations clearly indicated that during the (layer-by-layer) growth of the sputter deposited film, the Pt particles surrounded and covered the large Co clusters, composed of small fragments of Co-crystal, which could not contribute to XRD. 

**Table 1:** Particle sizes and lattice parameters of Pt particles.

Catalysts	Lattice Parameters (Å)	Crystallite Sizes (nm)	Interlayer distance (Å)
Pure Pt	3.92	7.28	2.26
Pt:Co (3:1)	3.90	7.23	2.25
Pt:Co (1:1)	3.89	7.29	2.24
Pt:Co (1:3)	3.83	4.82	2.21

**Figure 4:**
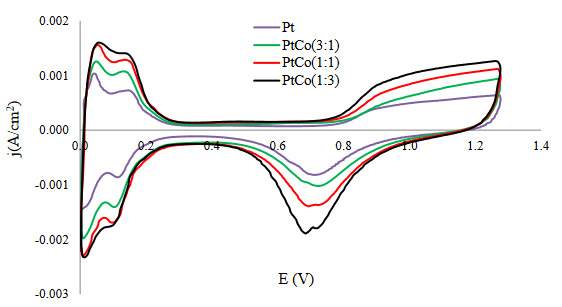
Cyclic voltammograms of sputtered Pt-Co layers in Ar-saturated 0.5 M H2SO4: potential scan rate 100 mV.s^-1^.

Two main factors that affect its electrochemical performance were the surface composition and the structure of the catalyst. CV responses of the sputtered Pt:Co films were presented in Figure 4. It could be seen that all CV curves had the shape of the pure Pt voltammogram which was well characterized in the literature [37], implying that the main active sites for the redox reactions were Pt atoms proceeding on the catalyst surface. Moreover, no anodic currents were observed due to the oxidation/dissolution of Co. This could be ascribed that the cobalt particles covered by noble metals were inaccessible to the electrolyte solution. The peaks between 0.04 V and 0.4 V vs. RHE, which belong to hydrogen adsorption/desorption were depicted for all samples. The current peak associated with the reduction of oxygen was in the region above 0.8 V vs. RHE. The cyclic voltammograms were used to calculate the electrochemically active surface area of the electrodes under the study, applying the well-established procedure of the integration of the area under the hydrogen adsorption/desorption peaks and using the value of 210 μC.cm^-2^ (the charge required for adsorption of hydrogen monolayer on 1 cm^2^ of smooth Pt electrode) as a correction factor [38]. 

The calculations showed that the EASA increases gradually with the increasing Co content, reaching the maximum value of 47.54 m^2^.g_Pt_^–1^ for Pt:Co (1:3). These results obviously indicated that the addition of Co influenced both the distribution of Pt particles and the size of Pt-Co particle. The Pt-Co particle size decreased with the reduction of Pt:Co ratio according to Table 1. As a result, the current peak related to the oxygen reduction reaction increased, and it reached the maximum value for the Pt:Co (1:3) sample, while the peak potential slightly shifted to the lower potentials. In the literature, several reports could be found about the catalytic activity towards hydrogen oxidation reaction. Their results were summarized and compared with this work in Table 2. Sigracet®39 BC and ELAT® LT 1400 W supported pure platinum catalyst exhibited similar and best activities in the table. However, it was worth noting that our work, which was prepared with sequential sputtering, showed remarkably enhanced activity compared with not only the pure platinum catalysts but also other bimetallic platinum-based electrocatalysts. 

**Table 2:** ECSA comparison of our work and literature

Support	Catalyst	Total Metal Loading(μg/cm2)	ECSA(m2/g)	Ref.
Sigracet39 BC	Pure Pt	127	58.9	43
ELAT LT 1400 W	Pure Pt	118	56.0	43
GDL-CT	Pure Pt	117	39.2	43
Vertically aligned CNF	Pure Pt	50	25.2	44
33 Å Ti sputtered GDL	Pure Pt	22	24.32	45
Ti sputtered E-TEK 120/30 WP	Pt-Ir(20%)	108	19.81	46
Ti sputtered E-TEK 120/30 WP	Pt-Ir(50%)	110	9.63	46
HOPG	Pt-Co	-	47	47
Sigracet 29 BC with microporous layer	Pt-Ni(25%)	22.19	9	48
Sigracet 29 BC with microporous layer	Pt-Ni(75%)	25.28	35	48
Freudenberg GDL(Our work)	Pt-Co(75%)	22.0	47.54	-

**Figure 5:**
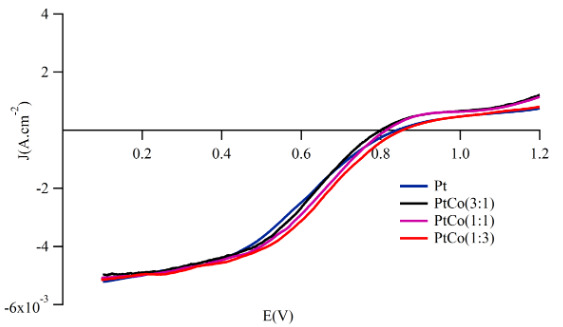
RDE polarization curves of sputtered Pt-Co catalyst layers in O2-saturated 0.5 M H2SO4 at rotation rate of 1600 rpm.

The electrocatalytic activity toward the oxygen reduction of the sputtered Pt-Co films was assessed by applying the method of linear sweep voltammetry and Koutecky–Levich plots based on equation. (1). Figure 5 showed the RDE polarization curves for all catalysts under the study. 

The RDE polarization curves with well-distinguished regions of kinetic, mixed, and diffusion limited reaction rate showed characteristic behavior reported in the literature for ORR on Pt in acid solutions [4, 39]. The process was governed by the charge transfer at very low overpotentials, then the ORR reaction proceeded under mixed diffusion-kinetic control in the range down to 0.75 V–0.5 V, after which the current started to level out reaching diffusion limited values at potentials of 0.25 V–0.2 V. The exchange current density, j_o_, was a qualitative measure for the intrinsic activity of the catalyst. In order to calculate j_o_, firstly construction of the mass-transfer corrected Tafel slopes was necessary, which, in turn, required determination of the kinetic current density. The common approach to calculate j_kin_, was to solve equation (1) regarding the kinetic current at different potentials and given rotating speed. This relation is known as a mass-transport correction for rotating disk electrodes 

(1)jkin=j.j1imj1im-j

where j_lim._ was the measured diffusion-limited current density, j_kin _was the kinetic current density, and j was the experimentally measured current density. 

**Figure 6:**
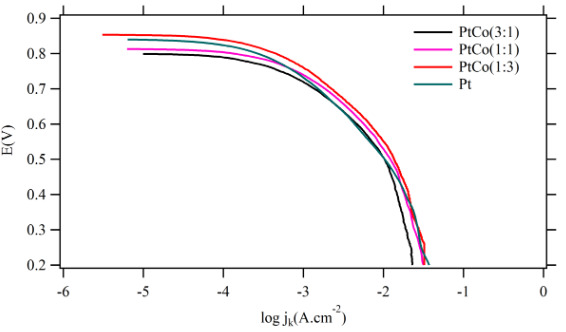
Mass transfer corrected Tafel plots for the Pt-Co samples under study.

The obtained mass transfer corrected Tafel plots were presented in Figure 6. Over 400 mV, the mass transfer corrected Tafel plots showed a wide linear range, which allowed determining the values of the Tafel slope and the apparent values of the exchange current density, j_0_^ap^. All determined results were summarized in Table 3.

**Table 3:** Kinetic parameters calculated from the mass transfer corrected Tafel plots.

Catalysts	EASA (m2/gPt)	joap (A.cm-2)	b (V/dec)	α	jo (A.cm-2)
Pt	23.75	1.12×10–9	0.131	0.48	2.90×10–8
Pt:Co (3:1)	32.50	2.72×10–10	0.128	0.33	8.86×10–9
Pt:Co (1:1)	37.71	5.86×10–10	0.130	0.26	2.21×10–8
Pt:Co (1:3)	47.54	3.99×10–10	0.128	0.26	2.30×10–8

**Figure 7:**
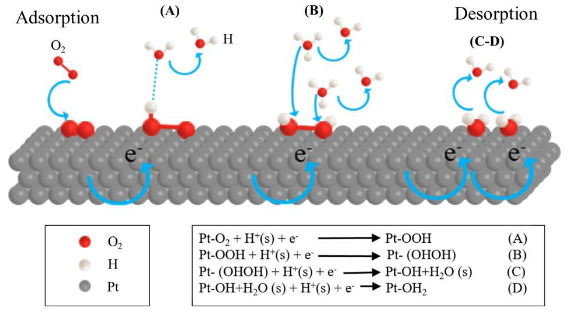
Possible ORR mechanism on the platinum surface and the equations.

As shown in Figure 6, a single Tafel slope were obtained for all samples with a value close to 120 mV.dec^–1^. This Tafel value indicated that rate-determining step of ORR was the first electron transfer reaction and given in Figure 7. Usually, different Tafel slope indicated the presence of oxides on the Pt surface, which was the reason for change of the ORR kinetics [40]. Since this was obviously not the case for the sputtered deposited ultra-thin Pt-Co catalytic films, the observed promotion of the ORR must have another origin. The values of the exchange current density j_o_ in Table 3 indicated a slight decrease in the intrinsic catalytic activity of Pt-Co compared to pure Pt but increase as the transition metal content increased. On the other hand, the polarization curves in Figure 5 demonstrated that the oxygen reduction was intense for the Pt:Co (1:3) sample, the catalyst that had the highest apparent exchange current density. The reaction mechanism and the rate-determining step of ORR were identical on both pure Pt and Pt-Co catalysts. This clearly emphasized that the potential dependent exponential terms and the chemical rate constant were basically same. Therefore, the pre-exponential coverage-dependent term must be responsible for the differences in the reaction kinetics, which were determined by the OH^-^_ad_ coverage. The higher activity for the ORR on Pt-Co could be ascribed to a lower O or OH coverage on Pt. In other words, the change in the occupancy of Pt 5d band increased the Pt electronegativity so the Pt-OH coverage get thinner, which caused a larger number of active sites for O_2_ reduction.

**Figure 8:**
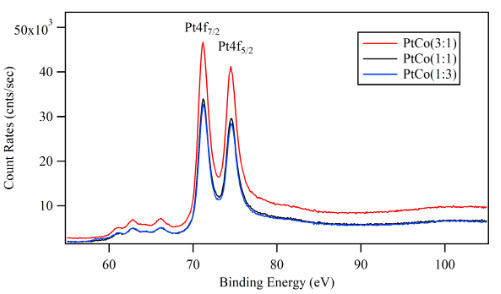
XPS spectra of the Pt4f of the sputtered Pt-Co nanocatalyst layers.

**Figure 9:**
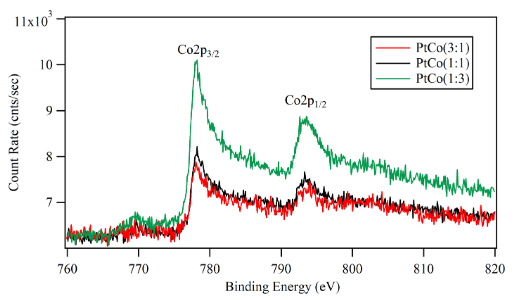
XPS spectra of the Co2p of the sputtered Pt-Co nanocatalyst layers: Co2p3/2 peak is shifted 0.1–0.3 eV higher binding energy compared to metallic Co (778 eV).

In order to clarify the increase in the ORR rate on the sputtered Pt-Co films, XPS analysis of the catalytic surfaces was performed. Figure 8 presented the XPS spectra of the Pt4f of the samples having different atomic ratios, and the Co2p peaks were shown in Figure 9. The experimental data were fitted with Voigt function after subtracting a background of the 6^th^-degree polynomial. The amount of Pt and Co on the surface were also calculated using the area under the corresponding peaks. The results were compared with the bulk atomic ratios calculated according to the TPP. 

The Pt4f_7/2_ peak in Figure 8 was located at 71.2 eV for all three samples. There was an indication of a shift in the binding energy relative to the standard value for pure Pt (71.0 eV) which implied an electronic interaction between Co and Pt atoms on the surface [41]. The shift on Pt4f_7/2_ peak did not change with Co content of Pt-Co films. In addition, the XPS spectra in Figure 9 showed some shift in the position of Co2p_3/2_ peaks about 0.1 eV to 0.3 eV compared to the standard value for pure Co (778.0 eV). The binding energy of Co2p_3/2_ was 778.1 eV for Pt:Co (3:1). For the samples with higher Co content such as Pt:Co (1:1) the Co2p_3/2_ was located at 778.2 and for Pt:Co (1:3) it appeared at 778.3 eV. However, the deviation of binding energy was much smaller compared to a previous study [42]. 

Another interesting result from the performed XPS analysis was the established difference between the calculated Pt:Co surface ratios and the bulk content of both metals determined according to the TPP formula. As shown in Table 4, although the Pt:Co surface ratio decreased consistently with the increase of Co content, all tree samples, especially Pt:Co (1:3) showed Pt-rich surfaces, which were opposite to what was expected. These observations indicated that Pt atoms could form a skin layer on Co cluster and screened the core electrons from Co atoms. 

**Table 4:** The bulk atomic ratios calculated according the TPP formula.

Catalysts	Pt Amount(μg/cm2)	Co Amount(μg/cm2)	Total Loading(μg/cm2)	Pt:Co Fit Results
Pt:Co (3:1)	20.0	2.0	22.0	7.27
Pt:Co (1:1)	17.0	5.1	22.1	5.11
Pt:Co (1:3)	11.5	10.4	21.9	2.40

As it was known from some other works [43-45], the power of the applied electric field and the gas pressure during the magnetron sputtering process had an essential effect on the particle size of the deposited film. In this work, the deposition pressure was kept constant at 1.2x10^‑3^ mbar and the power used for Pt and Co deposition were different (25 W and 15 W, respectively). In addition to applying different power for Pt and Co sources, the deposition rate for each sputtering cycle was also important. The timing of each source for one cycle was choose so that the thickness of each material are less than one-half monolayer. The applied higher power to Pt target and short sputtering cycles should result in a small Pt particle size, which claimed that Co clusters were capsulated by the smaller Pt particles and forming a core-shell type catalytic surface. This was the reason that the photoelectrons excited from the Co2p_3/2_ level interacted with Pt skin layer around Co core structure and lost some of its kinetic energy [41, 42]. This shift varied for the different ratios of Pt:Co layers starting from 0.1 eV to 0.3 eV for Pt:Co (3:1), Pt:Co (1:1) and Pt:Co (1:3) respectively. Otherwise, the surface energies of Pt and Co are comparable [46] and “layer by layer” deposition was expected.

Moreover the effect of core-shell type particle structure and electronic interaction between Pt and Co observed in XPS experiments showed as an improvement of catalytic reactivity. This improvement could be explained based on Norskov proposal in which the effect of decreased OH^-^ amount of Pt-Co catalysts was interpreted [47]. The surface reactivity significantly depended on the characteristics of the surface metal d-band. The d-band broadness of Pt could be changed due to the segregation of Pt on the large Co clusters, and the d-band center shifted in order to preserve the filling of the band. Previous DFT studies indicated that antibonding states were shifted down through the Fermi level due to compressive strain, thus weak bonding occurred. In contrast, tensile strain shifted up the antibonding states down through the Fermi level, and strong bonding occurred [48]. The electronic interaction between Co and Pt atoms on the surface could cause a downshift of the Pt d-band center conducted to the formation of occupied antibonding orbitals and it caused a weaker Pt-OH^-^ adsorption. This effect seemed more pronounced for the higher Co content.

In our work, Pt-Co catalysts had much higher activities than pure Pt catalyst even though the Pt loading of Pt-Co catalysts were lower compared to pure Pt sample. The total metal loading was fixed to 22 μg.cm^-2^ for all samples. The significant performance enhancement for ORR on electrodes made from Pt-Co core-shell like structured catalysts was related to the electronic interaction between Pt and Co.

**Figure 10:**
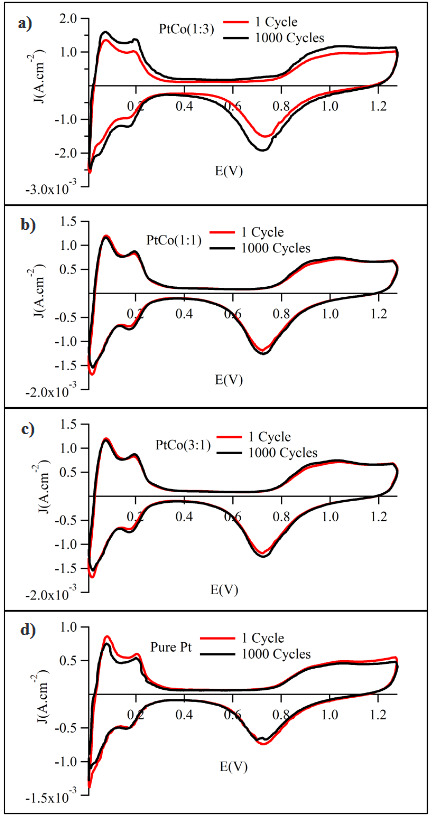
ADT experiments of sputtered Pt-Co catalyst layers; (a) Pt:Co (1:3), (b) Pt:Co (1:1), (c) Pt:Co (3:1), and (d) Pt

The Pt/C electrocatalysts was unstable for ORR, and this was one of the major problems with PEMFCs [3]. Under electrode potential cycling, dissolution of Pt catalysts caused loss of the electrochemical surface area. The stabilizing effect of Co on Pt was investigated by the accelerated durability testing (ADT). The accelerated durability test was performed by continuously applying potential sweeps from 0.00 V to 1.24 V to cause surface oxidation/reduction cycles of catalysts. The cycling voltammograms for this potential range before and after 1000 cycles are shown for all electrocatalysts in Figure 10. 

After 1000 cycles, pure Pt catalyst lost its electrochemical surface area almost 13%. It was well known that Pt atoms dissolved and re-deposited on the surface during cycles. This phenomenon would lead to agglomeration of Pt particles and loss of surface area [49-54, 55-57]. However, the addition of Co in the electrocatalysts increased the active surface area for all Pt-Co electrocatalyst after 1000 cycles. This effect seemed more pronounced for the higher Co content. Although in the literature, it was claimed that some bimetallic catalyst surfaces lost its activity with increasing test cycle our work showed that, the electrochemical surface area of Pt:Co (1:3) sample increased 11.48% after 1000 cycles. Durability comparison of this work and literature were summarized in Table 5. This result also showed that sequential cosputtering of Pt and Co enhanced the active surface area but also synthesized core-shell type bimetallic and highly durable clusters as a catalyst layer.

**Table 5:** Durability comparison of our work and literature.

Catalyst	Method	Initial ECSA(m2/g)	After ADT(% Durability)	Ref.
Pt/Sigracet®39BC (40 mg/cm2)	Chemical Reduction	72.4	–35	49
Pt/ELAT® LT 1400 W (40 mg/cm2)	Magnetron Sputtering	98.6	–10	49
PtCo/HOPG	Magnetron Sputtering	47	–36	53
PtNi(%50)/C (150 mg/cm2)	Powder Catalyst	31.2	–20	55
PtNi/HOPG	Magnetron Sputtering	36	–33	56
PtSn/C	Chemical Reduction	43	–37	57
Pt/ 1400 W ELAT® LT (410 mg/cm2)	Magnetron Sputtering	55.3	–20	58
PtY	Chemical Dealloying	53	–18.1	59
PtCo(75%)/FreudenbergGDL (40 mg/cm2) (Our work)	Magnetron Sputtering	47.54	+11.48	-

**Figure 11:**
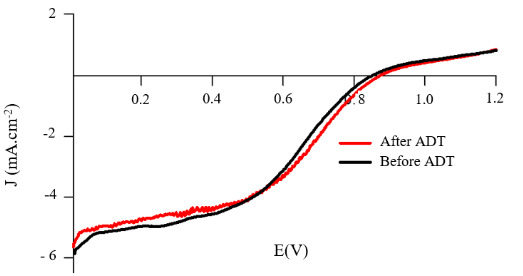
RDE experiment of sputtered Pt:Co (1:3) before and after ADT.

In order to clarify the dissipation of Co atoms on the surface, RDE polarization curves before and after ADT were presented in Figure 11. The half-wave potential of Pt:Co (1:3) after ADT shifts toward higher potential. Due to these potential shifts, electrocatalyst showed a significant increase in kinetic current at 0.9 V, and this obviously pointed out that Co did not dissolve during ADT. As we indicated above, Co particles were covered by noble metal, and thus were inaccessible to the electrolyte solution. Therefore, Co particles were not dissolved during potential sweeps. This result was supported by the XPS experiments done before and after ADT.

**Figure 12:**
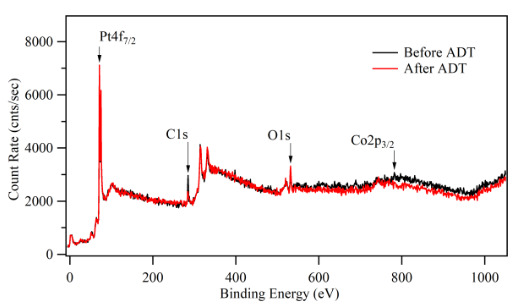
XPS survey spectra of sputtered Pt:Co (1:3) catalyst layers before and after ADT.

**Figure 13:**
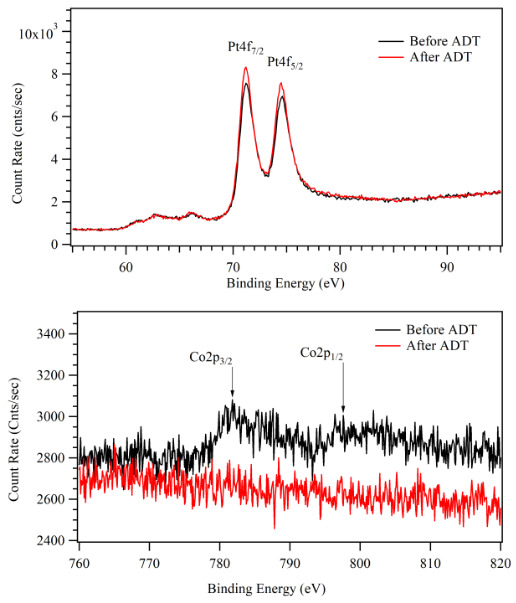
Pt 4f and Co 2p XPS spectra of sputtered Pt:Co (1:3) catalyst layers before and after ADT.

XPS analyses were performed for the Pt:Co (1:3) catalyst surface before and after ADT to observe the surface elemental composition changes due to the reactions. The survey of photo emission spectra and the high-resolution spectra of Pt4f and Co2p were shown in Figure 12 and Figure 13, respectively. The XPS result indicated that the elemental composition was the same before and after ADT but their ratio changed. After ADT, Pt4f intensity increased, but Co2p intensity decreased dramatically. This result showed that Pt skin layer thickness increased on the surface of the catalyst layer, covered Co clusters, and did not dissolve during ADT. The effect of Pt skin layer could be observed also as an improvement of the ADT result about 11.48% [53, 56]. 

**Figure 14:**
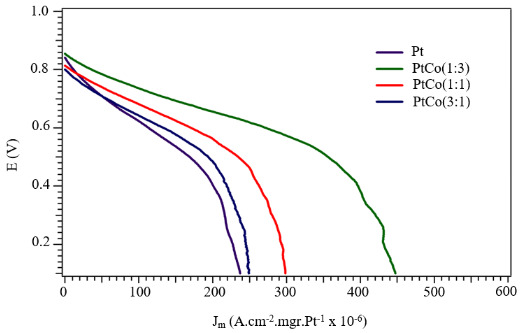
Mass activity jm (A.cm-2.mg.Pt-1) for the sputtered Pt-Co samples under study.

The amount of platinum catalyst decreased as the percentage of cobalt increased in the prepared electrodes while keeping the total metal loading constant. The mass activities of the catalysts were calculated by the ratio of the current density value obtained after the cell tests to the amount of platinum catalyst. As shown in Figure 14, the mass activity of the platinum catalyst is increased by increasing the amount of cobalt in the electrode layer and the highest mass activity is obtained with Pt:Co (1:3).

## 4. Conclusion

Magnetron cosputter deposition technique showed the possibility to fabricate highly active and cost-efficient Pt-Co composite catalytic films. According to XRD data, no peaks due to metallic Co or their oxides, and noticeable shift of Pt peaks toward high angle were observed. Moreover, addition of Co caused the shrink of Pt lattice in case of Pt:Co (1:3) sample and decreased the particle size because of strain caused by Co rich clusters. It was obvious that incorporation of Co as an interlayer resulted in an essential increase in both EASA with a maximum value of 47.54 m^2^.g_Pt_^–1^ for Pt:Co (1:3) and ORR activity of the cathode catalyst. The reason for the rising EASA was small Pt particles surrounding the larger Co clusters. Therefore, more Pt accumulated in the catalyst membrane interface. Moreover, the addition of Co leaded to increase in the intrinsic catalytic activity of the catalyst layer compared to pure Pt. Among the Pt-Co catalysts, the Pt:Co (1:3) had the highest apparent exchange current density, which was 2.3×10^–8 ^A.cm^–2^. This improvement in ORR efficiency was mainly due to the superior rising of the EASA. According to XPS studies, Co2p_3/2 _peak shifted 0.3 eV higher binding energy, which is related with the electronic interaction of Co and Pt. In addition, XRD and XPS results indicate that Pt particles surround the Co clusters. As a result, using sequential cosputtering of Pt and Co to prepare catalytic surfaces improved both the mass activity of Pt, ORR efficiency and durability of Pt-Co catalytic surfaces. 
